# IoT Technologies in Chemical Analysis Systems: Application to Potassium Monitoring in Water

**DOI:** 10.3390/s22030842

**Published:** 2022-01-22

**Authors:** José C. Campelo, Juan V. Capella, Rafael Ors, Miguel Peris, Alberto Bonastre

**Affiliations:** 1 Institute of Information and Communication Technologies (ITACA), Universitat Politècnica de València, Camino de Vera s/n, 46071 Valencia, Spain; jcapella@itaca.upv.es (J.V.C.); rors@itaca.upv.es (R.O.); bonastre@itaca.upv.es (A.B.); 2 Department of Chemistry, Universitat Politècnica de València, Camino de Vera s/n, 46071 Valencia, Spain; mperist@qim.upv.es

**Keywords:** smart sensor, ion-selective electrode, in-line monitoring, Internet of Things, water analysis, potassium determination, cloud services

## Abstract

The in-line determination of chemical parameters in water is of capital importance for environmental reasons. It must be carried out frequently and at a multitude of points; thus, the ideal method is to utilize automated monitoring systems, which use sensors based on many transducers, such as Ion Selective Electrodes (ISE). These devices have multiple advantages, but their management via traditional methods (i.e., manual sampling and measurements) is rather complex. Wireless Sensor Networks have been used in these environments, but there is no standard way to take advantage of the benefits of new Internet of Things (IoT) environments. To deal with this, an IoT-based generic architecture for chemical parameter monitoring systems is proposed and applied to the development of an intelligent potassium sensing system, and this is described in detail in this paper. This sensing system provides fast and simple deployment, interference rejection, increased reliability, and easy application development. Therefore, in this paper, we propose a method that takes advantage of Cloud services by applying them to the development of a potassium smart sensing system, which is integrated into an IoT environment for use in water monitoring applications. The results obtained are in good agreement (correlation coefficient = 0.9942) with those of reference methods.

## 1. Introduction

The Internet of Things (IoT) [1] is a concept that is being imposed at both social and scientific levels. It involves an ecosystem of intelligent objects along with services hosted in the cloud that have the ability to interact with each other and with their environment through the execution of computer programs that are interconnected using communication networks.

The main advantages of this new approach stem from the ubiquity of the processes involved (storage, calculation, etc.). Additionally, the availability of a wide variety of resources in the cloud (cloud services) offers many possibilities for the implementation of chemical analysis monitoring systems, notably increasing their versatility and performance without dramatically increasing the cost or complexity of the resulting system.

In some of our previous work [2,3], we focused on the research and development of techniques that allow for the implementation—by means of expert systems—of intelligent sensors that provide an increase in reliability and the rejection of interference from the point of view of efficiency, low cost, and reduced energy consumption.

Our paper integrates the advances in several research lines related to instrumentation and measurement issues. On one hand, many studies have covered Internet of Things measurements or IoT-based sensors [4,5,6]. Furthermore, IoT platforms and cloud processing environments have been addressed [7,8,9,10,11].

On the other hand, several research papers have focused on the processing of obtained data and the application of different algorithms to enhance the quality of the measurements [12,13].

Combining these research lines, our paper shows the benefits of the use of an IoT platform as an environment for IoT measurements obtained through ISE transducers. It is applied to chemicoanalytical problems, which arise from the need to monitor water quality in natural environments [14]. Among the parameters of interest, the main focus is on potassium, which can be found in drinking water as well as in vegetables, fruit, meat, bread, milk, and nuts. It plays an important role in the physical fluid systems of humans and assists in the functions of the nerves. When the kidneys do not work well, potassium accumulation can occur. This accumulation can lead to a disturbance in the heart rhythm and situations of weakness which, in very severe cases, can even result in respiratory paralysis.

On the other hand, along with nitrogen and phosphorus, potassium is one of the essential macronutrients for plant survival [15]. Its presence is of great importance for soil health, plant growth, and animal nutrition. Its primary function in plants is its role in maintaining osmotic pressure and cell size, thus influencing photosynthesis and energy production. This is why the element is required in relatively high proportions by developing plants. The consequences of low potassium levels are shown by a variety of symptoms: growth restriction, reduced flowering, less abundant harvests, and lower production quality. However, high levels of soluble potassium in water can damage germinating seeds, inhibit the uptake of other minerals, and reduce crop quality.

Water in rivers and lakes that is not in contact with carbonates will contain few dissolved ions (low mineralization water); in these cases, the concentration of potassium is as high as those of calcium, magnesium, and bicarbonate, chemicals which generally come from common rocks (aluminosilicates), either due to the action of washing with water or weathering produced by rain, wind, or atmospheric CO_2_ (carbonation). Therefore, the disintegration of rock in the presence of CO_2_ generates bicarbonates, and the different cations accompanying aluminosilicate emerge (usually K^+^ and Na^+^), thus enriching the water with these ions. For example, potassium feldspar weathering is one of the most important sources of K^+^ in natural waters:

3KAlSi_3_O_8_ + 2CO_2_ + 1 H_2_O ↔ 2K^+^ + 2HCO_3_^−^ + 6H_4_SiO_4_ + KAl_3_Si_3_O_10_(OH)_2_

In view of the above, it is easy to deduce that determination of the potassium level in natural waters (rivers, lakes, aquifers, etc.) is of great importance when carrying out a detailed follow-up of its evolution [16], given its effect on the water we use, the crops we raise, and the products of the latter. Emission spectroscopy techniques [17], such as flame photometry and inductively coupled plasma atomic emission spectroscopy (ICP-AES), are widely used for the analysis of potassium in water. Nevertheless, their high prices and the complexity of instrumentation required make them unsuitable for in-line monitoring, for which the utilization of ion-selective electrodes (ISEs) is much more advisable. The use of ISEs is widespread, and an increasing number of improvements to their design and performance are being made [18,19], mainly with the aim of increasing their sensitivity and selectivity (less interference). At the same time, work has been carried out to reduce their cost and energy consumption, factors which are of vital importance for their use in emerging technologies such as the Internet of Things.

Our approach integrates ISE-based devices into a standard IoT platform and provides a new architecture of services and algorithms that process the data in order to improve their quality, thus increasing reliability and providing metadata on the temporal and space evolution of the measurements.

The present paper reports the results of our current research to solve the proposed problem, which allows a step forward in chemical analysis monitoring systems to be taken. This solution, also applicable to other environments, allows the evolution from intelligent sensing nodes (information gathering) to intelligent sensing systems (systems that offer information wherever it is needed). The current proposal brings substantial benefits and is supported by the use of technologies that are already available and will see greater deployment in the future.

The article begins, in Section 1.1, by evaluating the utilization of IoT systems in analytical chemistry environments. From this, the need to rationalize and systematize the implementation of such system is detected. Therefore, Section 2 presents the architecture of a system for monitoring chemical parameters based on the IoT. This architecture is subsequently applied throughout Section 3 to a freshwater potassium monitoring application. For this purpose, Section 3.1 describes the IoT device and analytical devices (ISEs) used, whereas Section 3.2 discusses how these sensors transmit data to the cloud through the communication infrastructure. Then, Section 3.3 describes the data management services in the cloud that reject interference from other chemical species (Na^+^ and NH_4_^+^ are analyzed) and the redundant validation of the measurements obtained as well as the most closely related aspects. Finally, Section 4 presents the conclusions and actions that need to be carried out to produce future improvements.

### 1.1. Why Use the IoT in Analytical Chemistry?

Analytical chemistry, similar to other scientific disciplines, uses state-of-the-art technologies and services that integrate the IoT; consequently, it is necessary to clearly identify the technologies underlying this term, their principles (and what they can bring to analytical chemistry), and the relationships between them. In this article, these technologies are analyzed, classified by function, and organized in a hierarchical structure of levels that helps to improve the understanding of the relationships that exist between these technologies.

Moreover, many of these technologies have already been gradually integrated into the field of analytical chemistry, since they provide many advantages over more traditional technologies (instrumental techniques). These technologies carry out analyses that offer more reliable and precise data, thus providing more information. They also require the communication technologies that unite them to be improved.

Focusing on this last aspect, most nodes use wireless communication; hence, their energy consumption is a determining factor, since it limits the time for which they can operate without maintenance. In this sense, the latest trends have led to so-called low-power wide-area networks (LPWANs) with very little infrastructure that, using communication technologies with low bandwidth and energy consumption, reach high transmission distances. There are several options in this field, including Sigfox^®^ (Sigfox S.AF., Labège, France) [20] and its equivalent free (open) version LoRa^®^ [21], which is already fully operational and consolidated. Nevertheless, new proposals are emerging, such as NB-IoT^®^ [22] and, specifically, the installation of 5G telephony, whose medium-term prospects are very promising.

As a result of this constant technological evolution, in recent years, numerous ecosystems for the integration of hardware devices, embedded software, communication networks, and cloud processing, which are usually referred to as IoT platforms, have emerged. These modular IoT platforms are capable of incorporating new modules as new technologies appear.

It is very common to use cloud services to receive data from sensor networks. For example, some authors [7] have utilized CARDEA, a WiFi-based, assistive oriented “smart home” system, that is suitable for safety, security, automation, and monitoring purposes to improve the conditions of life for older adults. On the other hand, other researchers [8] have made use of network services to store and redirect data from sensor devices (back-end sensor layer) to end users (front-end layer). The same principle was employed in another study [9], with the particularity of using web services to offer users access to the data obtained through a PaaS (Platform as a Service) model. A similar function was carried out [10,11] with the Internet used to receive data from the sensors. In none of these examples was any correction algorithm applied to the measurements obtained. Three different designs have been proposed for an IoT-Enabled Power Monitoring system [12], but the only contribution of cloud computing is additional basic filtering of the reception and storage of data. Nevertheless, a preprocessing step is applied (to the individual data) in each of the nodes before sending the data to the cloud [13]. Furthermore, in the cloud, Artificial Intelligence techniques (a multilayer perceptron) are used for data processing. This last proposal is much more powerful than previous ones, but it has the disadvantage of not using an IoT platform itself, so therefore, it cannot be integrated with other IoT applications (which could provide contextual information to increase the value and quality of the data).

IoT platforms can be classified into two main groups: private platforms, whose incidence is marked by the company or group of companies that support them, and open platforms, whose origin is usually research projects or consortiums with the participation of public administration. Notable among the former are AWS from Amazon, Google Cloud from Google, ThingSpeak from MathWorks, Watson from IBM, and SmartThings from Samsung. Regarding the latter, in this proposal, we concentrate on FIWARE, which is described later.

In the literature, we found some contributions, most of them prototypes that, despite not being directly focused on chemical species analysis, have tried to evaluate some physicochemical parameters (temperature, flow rate, pH, turbidity…) in natural environments, such as rivers and lakes. In this way, interest in using this type of IoT system to provide greater functionality and allow for better monitoring of these variables to obtain more substantiated conclusions is rising. For this purpose, it is essential to choose one of the appropriate communication technologies to form part of the sensor node. Thus, a review [23] of the communication technologies that can be used for this task was performed, but it is outdated and should be updated with the most recent technologies. In [24,25], a precision irrigation system that relies on the LoraWAN protocol as an IoT infrastructure is described. A very interesting framework is proposed, with a subdivision into different layers for the development of monitoring systems in this field and an IoT platform, such as FIWARE, being used to manage the information collected.

In the study presented in [26], different parameters along a river, such as the pH, turbidity, temperature, and oxygen level, were monitored through a prototype system based on a microcontroller with a WIFI connection (unrealistic for real massive deployment in natural spaces) and stored in a database. Closer to reality from the point of view of the use of IoT technologies, although with a prototype character, is the proposal described in [27], in which Sigfox is used to store information on several river monitoring parameters in the cloud: turbidity, pH, dissolved oxygen, and temperature. Another approach that cannot be classified as an IoT system is that presented in [28], in which a probe is used to collect measurements—in water—of conductivity, dissolved oxygen content, pH, rhodamine, total algae, and turbidity. Once processed by a microcontroller, the data are sent through the GSM telephone network to a proprietary server to store and display the results. Following the same lines of use of GSM telephone connections, the water level of a river [29] can be monitored and sent to an IoT environment to which customers can subscribe. In the study presented in [30], the LoRa protocol and the Ericsson Cloud were used to establish an IoT framework. Due to the deployment area, i.e., Africa, other authors have also proposed [31] the use of GSM technology over other alternatives, such as Sigfox or LoRa, for reasons related to the necessary infrastructure. Interest in using other IoT technologies, such as LoRa or Sigfox, has been shown, but given the nature of the deployment to be carried out or the available infrastructure, authors have opted for the telephone connection.

However, it should be noted that none of the aforementioned approaches has made a contribution in the areas of treatment of the collected data, increase in the robustness of the measurements, detection of erroneous sensors, consideration of interference, or intelligent processing that provides robustness; the developed approaches merely dump the data provided by the sensors.

## 2. Proposal of an IoT Architecture for Chemical Analysis Systems

**Figure 1:**
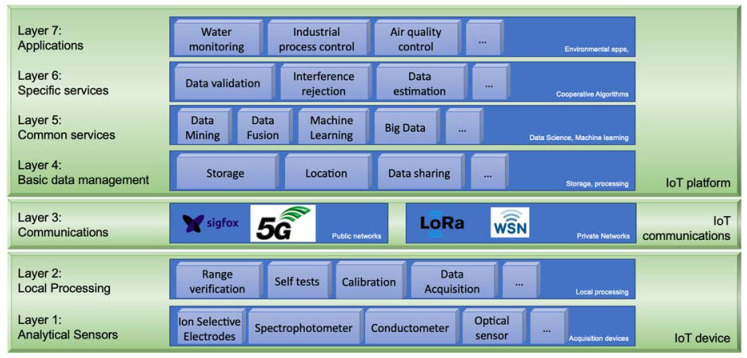
IoT services used in Analytical Chemistry and the relationships between them.

We are currently coping with a disparity of applications, services, platforms, and other elements comprising a complex ecosystem, which makes it difficult (if not impossible) for potential users of all these elements to understand and/or use them. Due to this complexity, in Figure 1, we propose an architecture that attempts to create a scenario where all of these elements can be located so that it is easy to understand what functions they perform and the relationships between them while favoring the development of complex applications that use combinations of them.

In this figure, each level represents a functionality that can be carried out fully or partially through different services, so that the functionalities of all services can be quickly defined based on their levels. As usual, the levels are organized hierarchically in such a way that each level uses the services offered by the lower level, performs certain actions (specific to its functionality), and offers, as a result of these actions, services at its higher level. For example, level 3 of the proposed architecture corresponds to communication services. At this level, the different communication infrastructures that can be used to satisfy the information transmission requirements within the system are shown. The available network will carry the data provided by level 2 services (data processed and offered by some IoT device/s at the origin) and deliver them to some element of the IoT platform at the destination (for example, a cloud storage service).

Within the proposed architecture, the bottom, included in what we have termed the IoT device, consists of what we now call a monitoring system for chemical analysis “based on ICT (Information and Communication Technologies)”. As shown, the system is composed of two levels:

**Level 1: Analytical Sensors.** This level is where the sensing elements are located (ISEs, spectrophotometers, optical sensors, etc.), with suitable transducers to convert certain parameters into signals that are proportional to the measured variable. These signals are processed by a computer system attached to the device. Initially, this constitutes a simple monitoring “node” (or device), which generates the measurements as digital information.

**Level 2: Local Processing**. Taking advantage of the capabilities of the computer system that incorporates the IoT device, the elements whose objective it is to locally improve the quality of the measurements 2 (such as self-testing, data formatting, or self-calibration techniques) are located in Level. For instance, as previously described, our research group has been working on some of these modules using artificial intelligence techniques that, along with redundancy in the sensor itself and with the addition of sensors for other species, allow more reliable and precise sensors to be obtained.

The bibliographic analysis presented in Section 1.1 shows that these monitoring devices may even include communication services to upload information processed onto a server to increase access to users of these systems. The emergence of computer networks and their generalization allows remote access to sensors. These communications are reflected in level 3 of the proposed architecture.

**Level 3: Communications**. At this level, all information transmission systems that could be used for remote access to information and management of the IoT devices are included. Although wired networks can be used, as shown in Figure 1, only wireless networks were considered due to their special characteristics. Within this level, we can also see that two types of wireless networks have been distinguished: public (such as Sigfox, NB-IoT and 5G) and private (such as LoRa). Paradoxically, public networks use proprietary technologies, while many private networks employ open technologies.

With the appearance of the IoT and the ambient intelligence (AmI) paradigm [32], an ecosystem of services [33,34] and a new concept regarding the structuring of information systems have arisen. For instance, in the monitoring systems of chemical analysis processes, in addition to the tasks carried out at level 2 in the device itself to improve its performance, it is possible to complement these tasks with others that, in this case, are executed in the cloud. This gives rise (Figure 1) to the IoT platform (later specified for potassium monitoring in rivers), the structure of which consists of the following levels:

**Level 4: Basic Data Management**. At this level, tasks related to the basic treatment of information are carried out. For example, information from IoT devices is processed and conveniently stored in the cloud. This was the final level in previous architectures, such as some of those described in Section 1.1.

At this level, storage in databases can be implemented to perform subsequent documentation, obtain data from other IoT applications, generate location metadata, and identify performing nodes. These data management results give very important added value to the information generated by the chemical analysis monitoring systems.

**Level 5: Common Services**. Software at this level retrieves the raw data provided by IoT devices from level 4 and processes them according to certain algorithms to obtain refined data. Numerous techniques may be applied for this type of processing, such as data mining, data fusion, and machine learning. This processing is common for all IoT platforms, regardless of the application. Likewise, its treatment can be invoked from higher levels once specific application-dependent processing has been applied.

**Level 6: Specific Services**. The common services at level 5 may be applied to many kinds of applications. In contrast, this is the level at which analytical chemistry would apply all necessary measures to treat chemical information to increase its added value. The starting point is a set of elaborated data (generated in Level 5), coming from either our IoT devices or from the cloud. From all of these data and the corresponding cooperative algorithms, which are especially suitable for analytical chemistry applications, new information offering extra value is obtained. Obviously, this information should be offered again in an open way so that other cooperative applications can use it in their algorithms.

At this level, other techniques can be implemented, namely, interference rejection, redundancy (for example, triple modular redundancy (TMR)), calibration and compensation, prediction of sensor degradation, validation with a reference method starting from other data sources in the cloud (metadata), monitoring of evolution curves against reference standards, and so on.

**Level 7: Applications**. Applications using the services of this architecture are located at this level. These applications, which interact directly with the end user, include river monitoring, industrial process control, and air quality monitoring.

The proposed architecture must be understood as a reference model. Regarding this concept and what is represented in Figure 1, it is worth highlighting the following:

– In each level, some elements belonging to that level have been placed as an example. The important thing is not those elements but the functionality common to all of them, which corresponds to the functionality of that level.
– For a particular application, there is no need to implement all levels or all services located on each level. It may even be necessary to incorporate additional services other than those shown in Figure 1.
– If a particular application is being studied, it is easy to know the functionality of each element by verifying only the level of Figure 1 on which it is located.

In the next section, an advanced chemical analysis system based on IoT technologies and its application to potassium monitoring is described following this architecture.

## 3. Potassium Monitoring System

In this section, an IoT-based potassium smart sensor using cloud services for monitoring the potassium concentration in fresh water is described as part of a system of water monitoring. This example is based on the deployment of several Lopy4 [35] nodes (IoT devices) that use the Sigfox infrastructure (IoT communications) to reach a FIWARE server (IoT platform). Each node is equipped with one or more ISEs for the determination of potassium or any of its interferents. These elements are briefly commented upon in the following sections.

The decision to use Sigfox came from the simplicity in the deployment of the nodes, since the company already provides the communication infrastructure with excellent coverage in the area. Lopy4 is an embedded microcontroller system that already incorporates this communication technology. Finally, the use of FIWARE as a platform, in addition to the aforementioned advantages, is conditioned by its use by local institutions.

**Figure 2:**
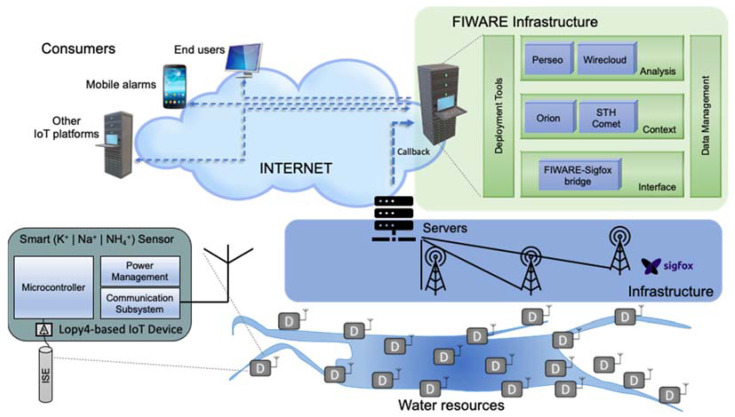
Potassium monitoring system.

Figure 2 shows the potassium concentration monitoring system, consisting of an IoT-based potassium smart sensor that uses cloud services.

Each of the platform components is described below with the help of the architecture proposed in Figure 1.

### 3.1. IoT Device

At the bottom of the proposed architecture in Figure 1 are the IoT devices. These constitute levels 1 and 2 of this architecture. For an IoT-based potassium smart sensor using cloud services, IoT devices based on ISEs have been developed, as shown at the bottom of Figure 2. Section 3.1.1 describes the part corresponding to the transducer (ISE) utilized, whereas Section 3.1.2 focuses on the rest of the elements that make up this device.

#### 3.1.1. Analytical Sensors

The “ISE” block is implemented within Level 1 “Analytical Sensors”. Potassium, sodium, and ammonium concentrations are measured by means of the corresponding homemade ISEs, which are fabricated and conditioned according to the method described in [36]. Stock solutions of KCl (for potassium), NaCl (for sodium), and NH_4_Cl (for ammonium) were obtained from a suitable amount of the corresponding reagent (analytical grade) using distilled and deionized water (specific conductivity < 0.2 µS cm^−1^). Thereafter, standard solutions were obtained by appropriate dilution. The characteristics of the ISEs are summarized in Table 1.

#### 3.1.2. Local Processing

Within Level 2 “Local Processing”, the “Data acquisition” block is implemented. To capture the information from the ISEs, a Lopy4-based IoT device (see Figure 2) was developed. This includes a microcontroller, a communication subsystem, and a power management unit.

Initially, the basic node can be configured with up to three ISEs. Some nodes include several different ISEs to improve interference rejection, with the concentrations of some types of interference being determined, while others use identical ISEs to increase fault tolerance. In this way, it is very easy to add new nodes to compensate for those ISEs that exceed their useful lifespans.

Lopy4 of Pycom was chosen as the microcontroller system. This development system offers the possibility of using various communication protocols, one of which is Sigfox. This makes it very desirable for the realization of prototypes and the evaluation, in the future, of other communication alternatives (LoRa, for example) without changing the sensing hardware.

The development system has a processing core based on the system on chip (SoC) ESP32 of the manufacturer Espressif [37], which has Tensilica Xtensa 32 LX6 [38] as the core processor. Additionally, it has digital and analog inputs and outputs. These analog channels have been used, after being treated by operational amplifiers [39], to capture the signal of the ISEs to be digitized. Once processed, this digital signal becomes relevant data that the microcontroller (integrated into the Sigfox infrastructure offers to the communication infrastructure) through the communication subsystem.

From Sigfox’s point of view, Lopy4 is certified as a Sigfox Class 0 device with a maximum transmission level of 14 dBm in Europe and a maximum range (in open space) of up to 50 km. Regarding consumption, levels of 12 mA in reception mode and 42 mA in transmission have been noted. In low-power mode (sleep mode), the consumption drops to 0.5 µA.

### 3.2. IoT Communication Infrastructure

The “Sigfox” block is implemented in Level 3 “Communications”. Within the different existing possibilities to implement the communication infrastructure of the IoT system for potassium monitoring, and given the characteristics of the application to be developed, we selected the Sigfox protocol [20], as this technology offers very good coverage and is widely spread.

For typical IoT applications in sensing environments, Sigfox offers the ability to send up to 140 messages per day (actually 144, but the last 4 are reserved for its internal protocol use) with a size of a dozen bytes at most. This is because, to comply with international telecommunications regulations, every user cannot occupy free bandwidth for more than 1% of each hour. This equates to six Sigfox 12-byte messages every hour, which is enough for most IoT application requirements, including ours.

### 3.3. IoT Platform

To complete the system description, we present the particularization (levels 4, 5, 6 and 7) that was carried out for the implementation of an advanced chemical analysis system based on IoT technologies applied to potassium monitoring in water. Given that, the services offered by the FIWARE platform have been used to implement these levels, we describe them before commenting level by level.

FIWARE is a platform promoted by the European Union for the development and global deployment of future Internet applications. It is a totally open, public, and free architecture. It provides a set of Application Program Interfaces (APIs) that facilitate application development. Additionally, open-source reference implementation of each of the FIWARE components is publicly available for users’ convenience.

**Figure 3:**
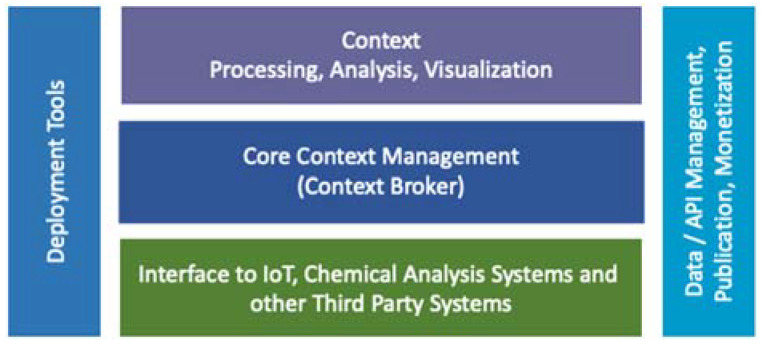
FIWARE architecture.

Figure 3 shows the FIWARE architecture, with a focus on the context broker.

The general structure of FIWARE is built around the “Context Manager General Enabler”, the only mandatory component in a FIWARE deployment. Its function is to manage the information in its context, allowing updates and offering access to the rest of the modules. Below this, there is an interface level with IoT systems, robots, and third-party systems, which receives information updates and translates the necessary updates.

The “Processing, data analysis and visualization” layer is located on the context manager, which allows the implementation of intelligent applications and other decision systems.

Covering the three levels, the “Context data/API management, publication, and monetization” module allows us to control the use of the data in terms of its dissemination and, where appropriate, its economic value.

Finally, the deployment tools, which also cover the three previous levels, permit the insertion and removal of modules in the system in the form of containers (Docker) that are directly importable from the FIWARE public repositories.

#### 3.3.1. Data Management and Cloud Services

Within Level 4 “Basic Data Management”, the “Storage” block is implemented by means of the STH-Comet component of the FIWARE platform, which provides the gateway to a database (based on mongoDB) where the results obtained from the IoT devices are stored.

For the monitoring of potassium in rivers, the FIWARE-Sigfox bridge module, published for free use in the FIWARE ecosystem, is employed at the interface level. This module implements the callbacks required to obtain the information collected by the sensors and that stored by Sigfox on its servers to offer it through the Orion Context Broker to the application modules.

In our case, the Orion Context Broker is used as the core of our FIWARE installation. As previously stated, it is in charge of managing the communications between information producers and consumers, the latter being expressed in the form of entities with attributes. Orion only manages the latest available values, which is why the aforementioned short-time historic entity, called Comet, is utilized, being placed in charge of storing the data permanently in a database.

Finally, the Orion Content Broker offers a tuple of values (potassium concentration, location, and time) in the cloud, which are available to all applications that subscribe, as shown in Figure 2. From this, it can be observed how other IoT platforms can access the cloud to obtain this information.

Regarding Level 5 “Common Services”, no service is implemented. This is an example of how, in the proposed architecture, it is not necessary to implement all levels.

#### 3.3.2. Specific Services and Potassium Monitoring Application

Following the above analysis of levels 4 and 5, we continue with the description of the proposed architecture and its application to potassium monitoring in water. Although the proposed architecture differentiates levels 6 and 7 in Figure 1, in Figure 2, both levels are implemented using FIWARE General Enablers [40] (Perseo and WireCloud), which, as shown in Figure 2, are run as services in the cloud. The complex event processing (CEP) module of FIWARE, called Perseo, allows us to specify treatment data by rules of behavior, while the generic enabler WireCloud offers a friendly graphical interface to the end user through web portals.

In Level 6 “Specific Services”, the “Interference Rejection” and “Data Validation” services are implemented. For this purpose, in this work, we propose the use of Perseo to implement both the TMR technique as a fault tolerance technique that is applicable to any application in general and interference rejection as a technique that is applicable to chemical analysis monitoring applications. On the other hand, WireCloud offers (through a friendly interface) potassium measurement itself along with its location as a concrete result of the proposed monitoring. The algorithms implemented in these services are detailed below:

“Interference Rejection” Block. In this application, an interference rejection service is implemented based on a mechanism that considers that the influence of the interfering agents on the measurement obtained can be compensated for by adjusting a straight line of regression, which can be applied to correct the obtained measurements when the interfering species are known. This algorithm is detailed in [2], where it was applied to a smart nitrate sensor and implemented by the Perseo module, which is included in the FIWARE platform.

The adjustment of the interference rejection mechanism was carried out through a series of experiments in which various potassium, ammonium, and sodium concentrations were combined, all of which were within the most frequent ranges in fresh water. Specifically, the most common potassium concentration values vary between 0.7 and 8.7 mg L^−1^. Regarding the interference species considered, the usual concentration of sodium is between 20 and 115 mg L^−1^, and that of ammonium is between 0.1 and 1.3 mg L^−1^.

To perform the measurements, all electrodes were simultaneously immersed in solutions (prepared by appropriate mixtures of the corresponding stock solutions) with the following resulting concentrations: (a) 0.70, 2.70, 4.70, 6.70, and 8.70 mg L^−1^ of potassium; (b) 20.0, 43.75, 67.50, 91.25, and 115.0 mg L^−1^ of sodium; and (c) 0.1, 0.40, 0.70, 1.00, and 1.30 mg L^−1^ of ammonium. The contents of K^+^, Na^+^, and NH_4_^+^ were then measured by combining these solutions in three ways, as shown in Table 2. It should also be highlighted that, in the area of Electroanalytical Chemistry, it is usually admitted that, when the ionic strength is under 0.01 M for monovalent ions (i.e., very dilute solutions), activities of the ionic species in the solution closely approach concentrations, so the use of concentration units (instead of activity) for measurements should not lead to significant error in the results obtained, even without the utilization of an ionic strength adjustment buffer. In the present work, ions occurring in the solutions employed were all monovalent and the ionic strengths of the most concentrated solution was 0.00186 M; that is why concentrations—and not activities—were utilized at all times (including calibration).

The corresponding regression analysis according to the procedure described in [3] produced the results presented in Table 3 and Table 4. This procedure consisted of the application of a linear regression of the measurements obtained in the previous experiments to calculate the coefficients of the following polynomial (1):

[K^+^]_estimated_ = m_1_ × [K^+^]_measured_ + m_2_ × [NH_4_^+^] _measured_ + m_3_ × [Na^+^] _measured_ + b (1)

As shown, the root-mean-square error is very low; thus, we can assume that the rejection works correctly. Although errors caused by interference are acceptable in this case, these techniques generally allow us to reject interference for ISEs with greater sensitivity to them.

“Data Validation” Block. In this block, and for the proposed application, a TMR system was implemented with the aim of increasing the reliability of the final measurement. To do this, we started with information from three different data sources. In our case, the measurements of three independent “sensors” were used, reaching level six. Taking advantage of the redundancy between measurements, self-correction methods capable of detecting anomalous measurements were utilized, thus increasing the precision of the measurements by averaging the sensor values. The word “sensors” is written in quotation marks to highlight the fundamental fact that it truly refers to information from any source (real sensor, data from other IoT platforms, etc.) that has passed through all levels of the architecture.

This information, which already has high added value (it is in the cloud, free from interference, conveniently stored, and available to applications), was reprocessed again to avoid errors due to sensor malfunction. For this purpose, the TMR module follows the algorithm proposed in [2,3], where the application of the TMR technique to environmental monitoring is described in detail. As a final result, the TMR offers us a single measurement that is immune to the malfunctioning of “real” sensors, since it either provides error-free measurement or indicates the existence of errors, which prevents erroneous decisions from being made (based on erroneous information); in this way, it is also possible to determine which sensor or sensors are giving erroneous results, with a view to their repair. Similar to the previous block, this functionality was implemented using Perseo.

Finally, since chemical analysis systems based on IoT technologies have been developed for potassium monitoring in water quality control applications, in Level 7 “Application”, the “Water Monitoring” block was partially implemented following the proposed IoT architecture. At this time, these potassium data are offered for either integration into this application or dissemination to other IoT entities that require them.

Figure 2 shows how different subscribers (mobile phone, tablet, personal computer, etc.) can access the cloud to obtain information from this module. This subscription is carried out through the Orion Context Broker, which is the standard module for data sharing.

### 3.4. Verification

In addition, validation of the results obtained by the three ISEs was carried out utilizing the corresponding official standard methods (see Table 1). To verify the system’s accuracy, an experiment was carried out to compare the measurements obtained by the proposed system—after all the previous steps —with a standard analysis method (ICP-AES) which served as a reference. For this purpose, samples were taken from a small dam (113 hm^2^) near the Mediterranean coast (coordinates: 40°01’56.3″ N 0°09’47.2″ W) at different points (suitably distributed) where nodes were located for subsequent analysis using the reference method. Water samples were then collected in small plastic bottles, properly sealed, and kept at 4 ºC before being analyzed in the laboratory.

Table 5 shows an example of the comparison between the measurements obtained at a given moment by our system against the values determined by the reference method [14] at the same time. It can be observed that both sets of values correlate well with each other (correlation coefficient = 0.9942).

## 4. Discussion

Following our research line on the automation of chemical analysis systems and the application of new technologies and processes that allow both increases in their reliability and precision and improvements in their simplicity and cost, we are currently working on the monitoring of chemical parameters in lakes and rivers and, more specifically, the evolution of the K+ concentration by using ISEs in these environments. The results of this research are gathered in this paper, where an intelligent potassium sensing system with interference rejection and fault tolerance is described. This system is integrated into an IoT environment, which allows us to take advantage of all of the benefits offered by cloud services.

Given the complexity of solutions, approaches, and emerging technologies capable of being used in the implementation of this monitoring system (and others similar to them in the field of analytical chemistry, as reflected in the literature review presented), the need to propose, in the first place, a generic architecture that rationalizes the systematic application of these solutions has emerged. This architecture is based on levels, where each level represents a functionality, which can be carried out totally or partially through different services so that the functionalities of all of them can be quickly defined on the basis of their levels. Furthermore, the levels are hierarchically organized so that a given level uses the services offered by the lower level, performs certain actions (specific to its functionality) and offers, as a result, services at the higher level. The great flexibility that this architecture provides should be noted, since, for systems implemented with it, it allows the following:

– The easy exchange of systems and/or services. For example, it is easy to change the communication network to another network that offers better performance and coverage, lower cost, or simply better availability in the place where the system will be deployed.
– The addition of new services (or the modification and/or removal of any of them). For example, a new module can be easily added if the system generates telephone alarms when the potassium concentration exceeds a certain threshold in a determined area.

All of this can be achieved with no changes to the rest of the system, since it is only necessary that the interfaces with the upper and lower layers are respected.

Following this architecture, the proposed sensing system is described. This consists of a set of IoT devices that, when conveniently deployed and through the ISEs available, collect the information to be measured. This information is introduced through a Sigfox communication network to the IoT platform. For this platform, FIWARE technology was selected, and through it, the different modules necessary to obtain the desired system characteristics were implemented (in this case, interference rejection and increased reliability through the TMR). Finally, the platform offers, upon subscription, information on the potassium concentration at different measurement points to interested users.

This work also highlights the ease of developing chemical analysis systems using the cloud services offered by current platforms (both communications and IoT). Such systems provide high added value, since the data are stored in the cloud and can be conveniently processed by its services and offered to both users and applications that require them.

In particular, with regard to the potassium sensing system developed for use in water, we must especially highlight major characteristics such as the fault tolerance of the system, which is achieved through the filtering of anomalous values and the use of the TMR technique. Likewise, it is also remarkable that it is possible to widely reject interference in the measurements with the ISEs as well as to carry out the simple deployment and replacement of nodes (regardless of the technology) thanks to the use of Sigfox.

In addition, the sensing system developed (using the new technologies available) offers a greater ease of deployment at a lower cost and with a greater integration capacity; this provides the opportunity to join other collaborative applications (which, by exchanging information with each other, can significantly increase the value chain of each component).

Finally, in terms of future research work (now in progress), we should take advantage of the fact that the generic architecture proposed will allow for the systematic application of new technologies to many other problems that frequently arise in the field of monitoring and control of chemical analysis processes, both environmental and other types.

## Figures and Tables

**Table 1:** Main features of the three Ion Selective Electrodes (ISEs) used.

ISE	Membrane Preparation	Calibration	Correlation with Official Method ^a^ (r^2^ Value)	Nernstian Slope (mV per Decade Change in Activity)	Limit of Detection (mg L^−1^ of Analyte)	Response Time (s)	Drift (mV h^−1^) in 0.01 M Standard Solutions
K^+^	tetrahydrofuran solution of 4% benzo-15-crown-5 crown ether, 28% polyvinyl chloride, and 68% orto-nitrophenylphenylether (plasticizer). Inner electrode solution: 0.01 M KCl	KCl standard solutions	0.9947 in the range 0.5–20.0 mg L^−1^	55 ± 4	0.4	<10	<0.5
Na^+^	4% ionophore, 65% dioctylphthalate, 30% polyvinyl chloride (PVC), and 1% potassium tetrakis(*p*-chlorophenyl)borate, in tetrahydrofurane (THF)	NaCl standard solutions	0.9972 in the range 1.0–200.0 mg L^−1^	55 ± 5	0.5	<10	<0.4
NH_4_^+^	400 mg of a mixture of 31% carboxylated polyvinylchloride, 4% nonactin, and 65% bis-(2-ethyl)hexyl sebacate in 5 mL of tetrahydrofurane (THF). Inner electrode solution: 10^−2^ M NH_4_Cl	NH_4_Cl standard solutions	0.9937 in the range 0.1–5.0 mg L^−1^	54 ± 5	0.02	<5	<0.4

^a^ The official methods used are inductively coupled plasma–atomic emission spectrometry (ICP-AES) for Na^+^ and K^+^ and UV-V spectrophotometry for NH_4_^+^.

**Table 2:** Design of the experiment.

		*[NH4^+^]* (mg L^−1^)				
		*0.10*	*0.40*	*0.70*	*1.00*	*1.30*
*[Na^+^]* (mg L^−1^)	*20.0*	*[K^+^] *(mg L^−1^)* = {0.70, 2.70, 4.70, 6.70, 8.70}*				
	*43.75*					
	*67.50*					
	*91.25*					
	*115.00*					

**Table 3:** Results of the linear regression.

**Coefficients**	**ISE Na^+^ (m_3_)**	**ISE NH_4_^+^ (m_2_)**	**ISE K^+^ (m_1_)**	**Term Independent (b)**
	−0.00038936	−0.15672332	0.96977118	0.16375884
Regression standard error	0.00015719	0.01283918	0.00191162	0.01755759

**Table 4:** Linear regression parameters.

**Correlation coefficient (r^2^)**	0.99953069
**F value**	85,902.0567
**Regression squared sum**	999.530693
**Standard error**	0.06227814
**Degrees of freedom**	121
**Residual sum of squares**	0.46930663

**Table 5:** Results obtained in the in-line monitoring of K^+^ in a small-size dam.

Sampling Point	[K^+^] mg L^−1^ (In-Line) ^a^	[K^+^] mg L^−1^ (Reference Method) ^b^
1	2.3	2.4
2	4.0	4.2
3	2.1	2.1
4	3.5	3.4
5	3.9	3.8
6	5.1	4.9
7	4.7	4.7
8	4.8	4.7

^a^ measured by the ISEs deployed at different points of the dam surface. ^b^ obtained off-line by inductively coupled plasma–atomic emission spectrometry (ICP-AES).

## Data Availability

Not applicable.
